# Organ-Restricted Hypereosinophilic Syndrome: A Case of Recurrent Hypereosinophilic Myopericarditis

**DOI:** 10.1016/j.jaccas.2025.105535

**Published:** 2025-09-26

**Authors:** Noora Alhajri, Samah Fadlelseed, Noha Abokhater, Shady Hegazi, Fulvio Salvo, Mohamed El Khashab

**Affiliations:** a Hospital Medicine Institute, Cleveland Clinic Abu Dhabi, Abu Dhabi, United Arab Emirates; b Department of Cardiology, Sheikh Shakhbout Medical City, Abu Dhabi, United Arab Emirates; c Heart and Vascular Institute, Cleveland Clinic Abu Dhabi, Abu Dhabi, United Arab Emirates; d Allergy and Immunology Institute, Cleveland Clinic Abu Dhabi, Abu Dhabi, United Arab Emirates

**Keywords:** acute heart failure, pericardial effusion, tamponade

## Abstract

**Background:**

Eosinophilic myocarditis (EM) is a rare inflammatory condition that is characterized by eosinophilic myocardial infiltration. We present a case of hypereosinophilic syndrome (HES) presenting as EM, complicated by cardiac tamponade.

**Case Summary:**

A 22-year-old man with a history of asthma presented with hypotension and chest pain. Work-up revealed myopericarditis with tamponade, eosinophil-rich pericardial effusion, and infection with influenza A. Despite initial improvement, he had recurrent episodes with elevated troponin, eosinophilia, and reduced left ventricular ejection fraction. Extensive work-up excluded malignancy and revealed biopsy-confirmed EM suggestive of HES. He responded to corticosteroids and was managed with guideline-directed heart failure medications.

**Discussion:**

Cardiac involvement as the initial presentation of HES is uncommon and represents a major contributor to morbidity and mortality.

**Take-Home Message:**

In patients presenting with acute heart failure and cardiac tamponade of uncertain etiology, a diagnostic work-up for EM should be initiated regardless of the presence of hypereosinophilia on the peripheral blood smear.

## Visual Summary

**Figure:**
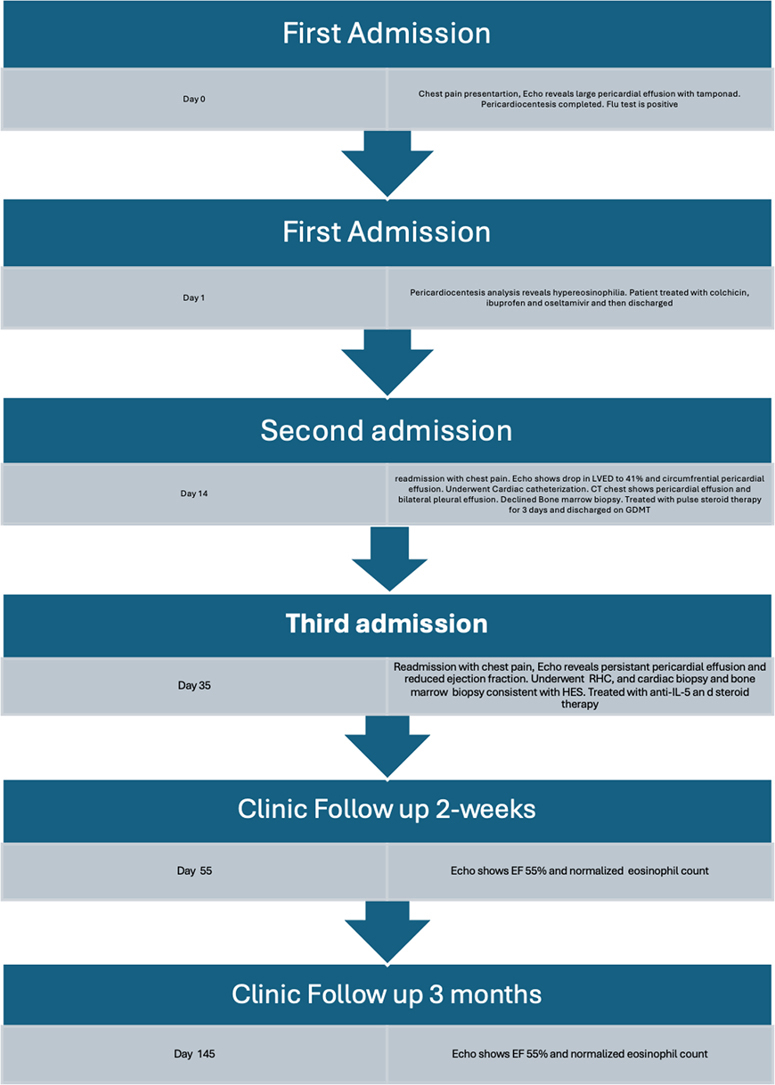

## History of Presentation

**Figure 1:**
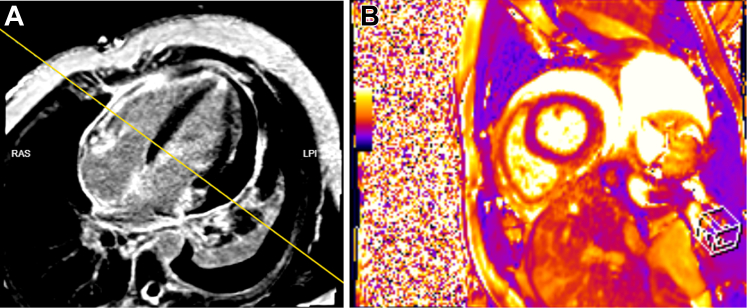
Cardiac MRI at Initial Presentation Cardiac MRI with contrast T2 phase showing mid global LV and RV hypokinesia with asynchronous LV contraction and mild hypokinesia of the midventricular anteroseptal segment. The LV ejection fraction was 45%, and the RV ejection fraction was 43%. An ill-defined LGE in the basal inferior and inferolateral segment with myocardial edema affecting these segments was suggestive of myopericarditis. Moderate pericardial effusion can also be seen. LGE = late gadolinium enhancement; LV = left ventricle; MRI = magnetic resonance imaging; RV = right ventricle.

A 22-year-old physically active man with a history of asthma presented to an outside hospital after 1 day of left-sided chest pain associated with dyspnea and dizziness. On physical examination, his blood pressure was 81/56 mm Hg, heart rate was 125 beats/min, and respiratory rate was 22 breaths/min. Oxygen saturation and body temperature were normal. Laboratory results are summarized in Table 1. Electrocardiogram (ECG) showed sinus rhythm with T-wave inversions in leads V_2_ to V_4_. An influenza A test was positive. Transthoracic echocardiogram revealed a large circumferential pericardial effusion (2.7 cm on the apical window) with evidence of tamponade physiology, including right ventricular diastolic collapse. Emergent pericardiocentesis was performed, which was significant for elevated eosinophils, accounting for 69.9% of cell differentials, whereas the culture and cytology of the pericardial effusion were negative. Cardiac magnetic resonance imaging showed mild global reduction in left ventricular (LV) ejection fraction (45%), with ill-defined subepicardial and midwall late gadolinium enhancement in the inferior and inferolateral walls, consistent with acute myopericarditis (Figure 1). The initial impression was myopericarditis due to influenza A virus, and the patient was treated with 0.6 mg colchicine, ibuprofen, and oseltamivir phosphate and was discharged.

**Take-Home Messages**

• The diagnosis of hypereosinophilic syndrome is complex and necessitates a multidisciplinary approach for accurate identification and management.
• In patients presenting with acute heart failure and cardiac tamponade of uncertain etiology, a diagnostic work-up for eosinophilic myocarditis should be initiated regardless of the presence of hypereosinophilia on the peripheral blood smear at presentation.

**Table 1:** Laboratory Results at Corresponding Admissions and Follow-Up Dates

Date and Laboratory Test	Result
First admission: December 12, 2024	
Troponin T	0.31 μg/L
NT-proBNP	796 pg/mL
WBC	25.5 × 10^9^/L
Eosinophil count	10.4 × 10^9^/L
CRP	17 mg/L
Second admission: January 7, 2025	
Troponin T	1.102 μg/L
NT-proBNP	6,779 pg/mL
Eosinophil count	7.8 × 10^9^/L
Third admission: January 31, 2025	
Troponin T	2.37 μg/L
NT-proBNP	1,947 pg/mL
WBC	23.1 × 10^9^/L
Eosinophil count	12.19 × 10^9^/L
AST	19 U/L
ALT	41 U/L
CRP	28.1 mg/L
B_12_	374 pmol/L
Creatinine	61 μmol/L
Clinic follow-up at 2 wk: February 21, 2025	
Troponin T	—
NT-proBNP	1,268 pg/mL
WBC	15 × 10^9^/L
Eosinophil count	0.1 × 10^9^/L
Clinic follow-up at 3 mo: May 6, 2025	
NT-proBNP	186 pg/mL
WBC	8.9 × 10^9^/L
Eosinophil count	0.1 × 10^9^/L

ALT = alanine aminotransferase; AST = aspartate aminotransferase; CRP = C-reactive protein; NT-proBNP = N-terminal pro–B-type natriuretic peptide; WBC = white blood cells.

## Medical History

The patient was an active smoker (2.5 packs/year) who worked in the armed forces. He had a childhood history of bronchial asthma and a remote history of congenital pyloric stenosis that was repaired when he was 20 days old. He had no history of atopic dermatitis or nasal polyps. He had no family history of autoimmune disease.

## Differential Diagnosis

The diagnosis of eosinophilic myocarditis (EM) was speculated based on the evidence of eosinophil-rich pericardiocentesis. However, further characterization of the etiology was needed, as the initial impression was reactive EM due to influenza A. Other differential diagnoses included organ-restricted hypereosinophilic syndrome (HES), giant cell myocarditis, eosinophilic granulomatosis with polyangiitis (EGPA), parasitic infection, and malignancy.

## Recurrent Presentation and Multidisciplinary Team Approach

One week after discharge, the patient returned to an outside facility with recurrent chest pain, dyspnea, and markedly elevated eosinophil count (Table 1). ECG showed ST-segment depressions with T-wave inversions in leads I, II, III, aVL, and V_4_ to V_6_. Echocardiography demonstrated mild to moderate circumferential pericardial effusion (1.8 cm) with no evidence of tamponade physiology, borderline dilation of the inferior vena cava with reduced collapse stability, and impaired LV systolic function (LV ejection fraction: 41%). Pan–computed tomography scan showed mild pericardial effusion, congestive hepatomegaly, bilateral pleural effusions, bilateral lower lobe consolidation, mild pulmonary edema, and no evidence of lymphadenopathy. He underwent cardiac catheterization, which revealed angiographically normal coronary arteries. He was evaluated by the hematology and infectious disease teams for advanced eosinophilia work-up including possible genetic testing and bone marrow biopsy to rule out bone marrow malignancies, however the patient declined. He was started on pulsed steroid therapy 1,000 mg for 3 days, received empiric antibiotic treatment with piperacillin-tazobactam, and decontamination therapy for possible parasitic infection with albendazole and ivermectin given positive *Toxocara* IgG levels in the past, however repeated testing was negative.

The patient was discharged on bisoprolol 2.5 mg once daily, colchicine 0.6 mg twice daily, eplerenone 25 mg once daily, ivabradine 5 mg twice daily, empagliflozin 10 mg once daily, and sacubitril-valsartan 49/51 mg once daily. The impression was acute myopericarditis after influenza A infection, complicated by acute heart failure with moderately reduced ejection fraction.

## Disease Progression and Advanced Investigations

**Figure 2:**
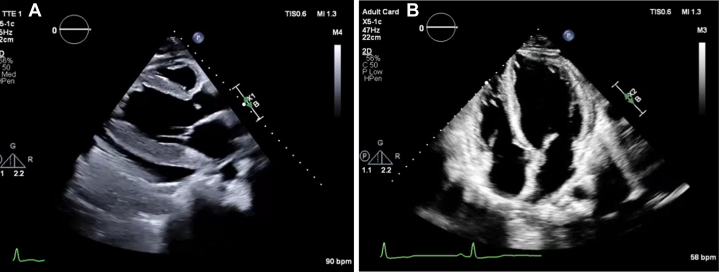
Transthoracic Echocardiography During Third Hospital Admission (A) Parasternal long-axis view showing moderate posterior and lateral pericardial effusion adjacent to the LV measuring 1.6 cm, with no RA/RV collapse. The LV is mildly dilated, LV systolic function is mildly decreased, EF is 44% ± 5%, with global hypokinesia of LV. The LVEF was visually estimated as indicating mild to moderate LV systolic dysfunction. LV diastolic function was indeterminate. (B) Apical 4-chamber view after treatment shows normal-sized LV. The LV systolic function was mildly decreased, EF was 50% ± 5%, LV diastolic function was indeterminate. The right ventricle was normal in size, RV systolic function was normal, and estimated RV systolic pressure was 22 mm Hg, consistent with normal pulmonary artery pressures. There is a small circumferential pericardial effusion measuring 0.6 cm predominantly anterior to the RV. EF = ejection fraction; LV = left ventricle; RA = right atrium; RV = right ventricle.

Three weeks after the second discharge, the patient returned to the outside hospital with recurrent chest pain and dyspnea. Assessment of his vital signs revealed blood pressure of 91/68 mm Hg, heart rate of 110 beats/min, respiratory rate of 24 breaths/min, and temperature of 36.6 °C (Table 1). ECG revealed sinus tachycardia with a premature atrial contraction. A reduction in ejection fraction with posterior LV hypertrophy and wall motion abnormality was notable on transthoracic echocardiography (Figure 2). The right ventricle was normal in size; however, function was mildly decreased with an estimated right ventricular systolic pressure of 36 mm Hg, consistent with mild pulmonary hypertension, and a moderate pericardial effusion measuring 1.6 cm was noted. The patient was immediately started on methylprednisolone 1 mg/kg every 12 hours for 3 days followed by 1 mg/kg daily and was transferred to our hospital for continuity of care.

**Figure 3:**
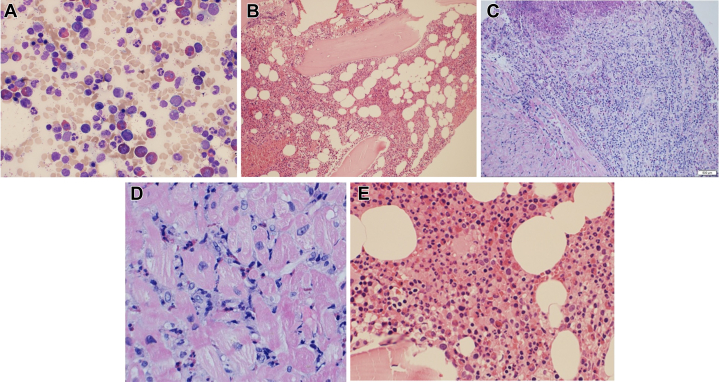
Results of Endomyocardial Biopsy During Third Hospital Admission (A) Peripheral blood smear demonstrating marked hypereosinophilia. Numerous eosinophils with characteristic bilobed nuclei and abundant bright eosinophilic (red-pink) cytoplasmic granules are seen diffusely scattered throughout the field. (B) Bone marrow aspirate showing hypercellular marrow with markedly increased eosinophilic precursors and mature eosinophils interspersed among myeloid and erythroid elements. (C) Low-magnification cardiac tissue biopsy stained with May-Grunwald Giemsa showing increased proportion of mature eosinophils accounting for 22.2% without any dysplastic features. (D) High-magnification cardiac tissue biopsy shows a diffuse inflammatory infiltrate throughout the myocardium. (E) Bone marrow biopsy demonstrating markedly increased eosinophilic precursors and mature eosinophils with abundant eosinophilic cytoplasmic granules and bilobed nuclei. The marrow is hypercellular with reduced adipose spaces and preserved trilineage hematopoiesis.

A right heart catheterization with endomyocardial biopsy was performed and indicated a mean pulmonary capillary wedge pressure of 32 mm Hg with a cardiac index (Fick method) of 3.32 L/min/m^2^. The biopsy (Figure 3) showed myocyte hypertrophy with multifocal inflammatory infiltrates composed of lymphocytes, macrophages, and abundant eosinophils in the perivascular, interstitial, and endocardial locations. A bone marrow biopsy and aspirate revealed hypercellular marrow with significant eosinophilia, accounting for 22% of the cell differential. The eosinophils were mostly mature forms without dysplastic features, favoring reactive eosinophilia. Flow cytometry on the bone marrow aspirate was negative for increased blasts, abnormal T-cells, or clonal B lymphocytes. An eosinophilia FISH (fluorescence in situ hybridization) panel was negative for gene rearrangements. The findings of EM with persistent peripheral and bone marrow eosinophilia, absence of dysplasia, negative mutations, presence of clonal T-cell population, and exclusion of secondary causes (EGPA, drug reaction with eosinophilia and systemic symptoms [DRESS], parasites) strongly supported lymphocytic-variant HES.

## Management

Given the inconclusive results from the hematologic and infectious disease work-ups, consultation with the immunology and allergy teams was pursued for further evaluation. The patient was also started on monoclonal antibody (anti-interleukin 5) mepolizumab 300 mg every 4 weeks in view of disease recurrence, and he continued on a prednisolone taper regimen in addition to guideline-directed medical therapy for heart failure, including bisoprolol 2.5 mg once daily, eplerenone 50 mg once daily, ivabradine 5 mg twice daily, empagliflozin 10 mg daily, and sacubatril-valsartan (100 mg) 49 to 51 mg twice daily. Repeat echocardiography before discharge showed an LV ejection fraction of 44%. The peripheral blood eosinophil count decreased significantly from a peak absolute eosinophil count of 12.97 × 10^9^/L before immunosuppressive therapy to 0.01 × 10^9^/L.

## Follow-Up

The patient was seen in clinic 2 weeks after discharge and was asymptomatic. A repeat echocardiogram showed improvement of ejection fraction to 50%, with normalization of the LV global strain that was seen in the previous images. Repeat absolute eosinophil count was 0.01 × 10^9^/L, and repeat N-terminal pro–B-type natriuretic peptide was 1,268 pg/mL.

## Discussion

EM is a rare inflammatory condition that is characterized by eosinophilic myocardial infiltration and a state of peripheral eosinophilia.^1^, ^2^, ^3^ Although the presentation of EM without peripheral eosinophilia has been documented in the literature,^4^ the clinical spectrum of disease presentation ranges from mild to a fulminant necrotizing EM.^5^, ^6^, ^7^ Studies have shown that during the hypereosinophilic state, toxic eosinophilic granule proteins, specifically major basic protein 1 (MBP1), deposit in the myocardium, and the eosinophil peroxidase impair the anticoagulation effect of thrombomodulin and activate platelet aggregation, leading to thromboembolic events.^5^^,^^6^ The association between eosinophilia and myocardial injury is well documented in the literature^8^^,^^9^ and is outlined in Table 2. We present a case of HES presenting as EM complicated by cardiac tamponade. Cardiac involvement as the initial presentation of HES is uncommon and represents a major contributor to morbidity and mortality.^1^, ^2^, ^3^, ^4^, ^5^ Hypereosinophilia is defined as an eosinophil count of >1,500/mL. The diagnosis of HES, per the fifth edition of the revised World Health Organization and International Consensus Classification of eosinophilic disorders,^10^ must include all the following criteria:

1. Sustained eosinophilia >1.5 × 10^9^, ≥10% eosinophils on 2 examinations, 1 month apart;
2. Percentage of eosinophilia on bone marrow biopsy exceeding 20% of all nucleated cells;
3. End-organ damage attributable to tissue infiltration by eosinophils;
4. Exclusion of reactive eosinophilia, lymphocytic-variant HES, and other myeloid neoplasms.

**Table 2:** Results From Infectious Disease, Immunological, and Hematological Work-Ups

Infectious Disease Work-Up	Result	Immunological Work-Up	Result (Reference)	Hematological Work-Up	Result
HIV	Negative	IgE	821 (≤100.0)	Leukemia/lymphoma karyotype	Normal male 46 XY
Strongloides	Negative	Anti–double strand DNA	<1 IU/mL (<4 IU/mL)	Cytogentics	Normal
Schistosoma, *Toxocara*, amebiasis	Negative	Antinuclear antibodies	Negative (negative)	BCR/ABL	Negative
Stool parasite examination	No ova or parasite	p-ANCA (MPO)	1:20 titer (<1:20 titer)	Pathology flow cytometry	CD 3: 5.2%, CD 4: 3.1%, CD 8: 1.5%, CD 19: 0.35%, eosinophils: 41.4%
Parvovirus B19	IgG positive, IgM negative	c-ANCA (PR3)	1:20 titer (<1:20 titer)	T-cell rearrangement	Normal
Toxoplasmosis	IgG negative, IgM negative	Rheumatoid factor	41 IU/mL (<13 IU/mL)	5q32 (PDGFRB sep)	Normal
Coxsackie A	IgM negative, IgG negative	IL-5	0.2 pg/mL (<1.5 pg/mL)	9p24.1 (JAK2 sep)	Normal
EBV	IgG positive, IgM negative	Tryptase	Normal (—)		
CMV	IgG positive, IgM negative	FIP1L1-PDGFRA	— (—)		

ANCA = antineutrophil cytoplasmic antibodies; CD = clusters of differentiation; CMV = cytomegalovirus; EBV = Epstein-Barr virus; IL = interleukin; PDGFRA = platelet-derived growth factor receptor A.

Guidelines for diagnosing EM include the following^7^:

1. Elevated eosinophil count >500/mL;
2. Elevated level of cardiac enzymes such as troponin T or creatine kinase–MB;
3. ECG changes;
4. Cardiac symptoms such as chest pain, dyspnea, and palpitations;
5. Echocardiographic evidence of wall motion abnormality or asynergy;
6. Endomyocardial biopsy with eosinophilic infiltration of tissue.

The patient met these diagnostic criteria, with evidence of organ damage restricted to the heart, hence the diagnosis of EM secondary to HES was made.

Evidence regarding treatment of EM is scarce. A 2017 systematic review by Brambatti et al^1^ suggested that corticosteroids can reduce the mortality rate in EM and emphasized the importance of identifying the etiology of EM to determine the optimal treatment plan; for instance, albendazole for *Toxocara canis*–associated EM, cyclophosphamide for EGPA-associated EM, and imatinib in the myeloproliferative variant of the PDGFRA (platelet-derived growth factor receptor A)–associated variant of HES. Although the treatment of EM is guided by its etiology, it is important to reinforce the need for clinical trials to test the efficacy of immunosuppressive therapies.

## Conclusions

We identified an unusual case of recurrent EM presenting as cardiac tamponade, with an acute drop in LV ejection fraction and progression of heart failure despite initial corticosteroid therapy. This report highlights the need for clinical guidelines for the management of EM. International registries and clinical trials are needed to improve overall morbidity and mortality.

**Visual Summary:**
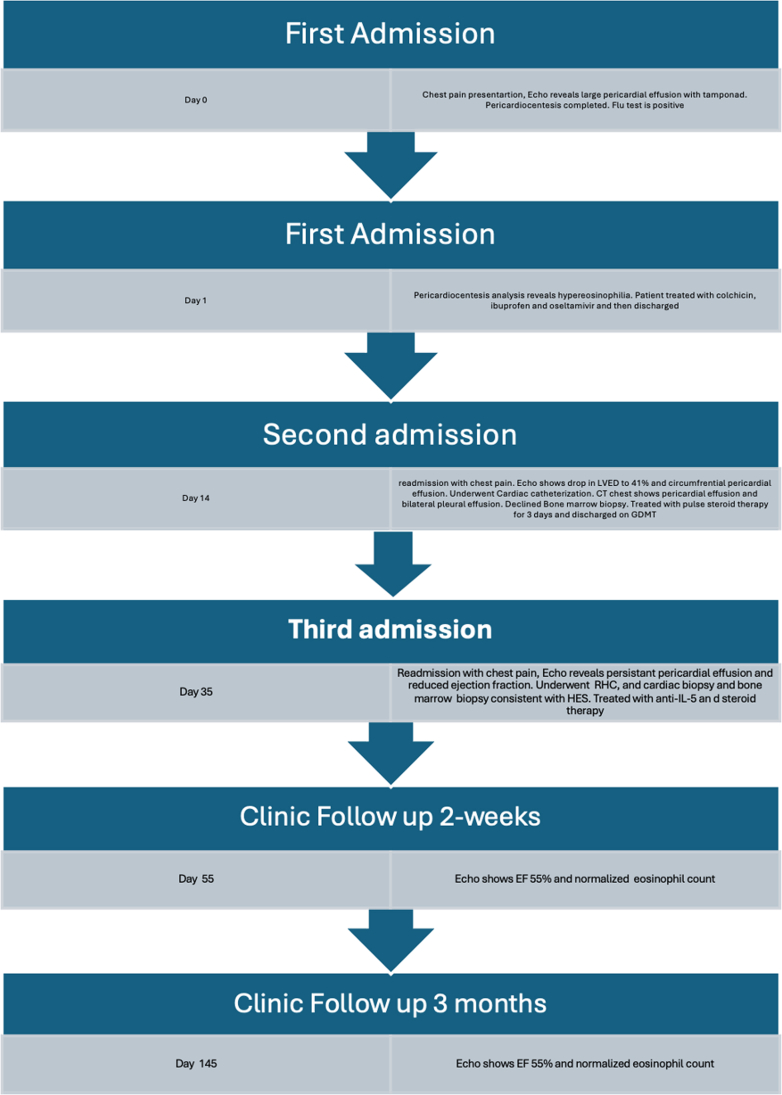
Timeline of Case Presentation and Follow-Up CT = computed tomography; EF = ejection fraction; HES = hypereosinophilic syndrome; GDMT = guideline-directed medical therapy; RHC = right heart catheterization.

## Funding Support and Author Disclosures

The authors have reported that they have no relationships relevant to the contents of this paper to disclose.

## References

[1] Brambatti M., Matassini M.V., Adler E.D., Klingel K., Camici P.G., Ammirati E. (2017). Eosinophilic myocarditis: characteristics, treatment, and outcomes. J Am Coll Cardiol.

[2] Cheung C.C., Constantine M., Ahmadi A., Shiau C., Chen L.Y.C. (2017). Eosinophilic myocarditis. Am J Med Sci.

[3] Ammirati E., Moslehi J.J. (2023). Diagnosis and treatment of acute myocarditis: a review. JAMA.

[4] Lee J.-Y., Lee S.H., Kim W.H. (2023). Fulminant eosinophilic myocarditis without peripheral eosinophilia. Tex Heart Inst J.

[5] deMello D.E., Liapis H., Jureidini S., Nouri S., Kephart G.M., Gleich G.J. (1990). Cardiac localization of eosinophil-granule major basic protein in acute necrotizing myocarditis. N Engl J Med.

[6] Wright B.L., Leiferman K.M., Gleich G.J. (2011). Eosinophil granule protein localization in eosinophilic endomyocardial disease. N Engl J Med.

[7] Ogbogu P.U., Rosing D.R., Horne M.K. (2007). Cardiovascular manifestations of hypereosinophilic syndromes. Immunol Allergy Clin North Am.

[8] Kleinfeldt T., Nienaber C.A., Kische S. (2010). Cardiac manifestation of the hypereosinophilic syndrome: new insights. Clin Res Cardiol.

[9] Parrillo J.E. (1990). Heart disease and the eosinophil. N Engl J Med.

[10] Gotlib J. (2017). World Health Organization-defined eosinophilic disorders: 2017 update on diagnosis, risk stratification, and management. Am J Hematol.

